# HR1 Robot: An Assistant for Healthcare Applications

**DOI:** 10.3389/frobt.2022.813843

**Published:** 2022-02-07

**Authors:** Valentina Vasco, Alexandre G. P. Antunes, Vadim Tikhanoff, Ugo Pattacini, Lorenzo Natale, Valerio Gower, Marco Maggiali

**Affiliations:** ^1^ iCub Tech, Istituto Italiano di Tecnologia (IIT), Genoa, Italy; ^2^ Human Sensing and Perception, Istituto Italiano di Tecnologia (IIT), Genoa, Italy; ^3^ IRCCS Fondazione Don Carlo Gnocchi, Milan, Italy

**Keywords:** humanoids, assistive robots, human-robot interaction, healthcare, autonomous systems, timed up and go

## Abstract

According to the World Health Organization^1,^ ^2^ the percentage of healthcare dependent population, such as elderly and people with disabilities, among others, will increase over the next years. This trend will put a strain on the health and social systems of most countries. The adoption of robots could assist these health systems in responding to this increased demand, particularly in high intensity and repetitive tasks. In a previous work, we compared a Socially Assistive Robot (SAR) with a Virtual Agent (VA) during the execution of a rehabilitation task. The SAR consisted of a humanoid R1 robot, while the Virtual Agent represented its simulated counter-part. In both cases, the agents evaluated the participants’ motions and provided verbal feedback. Participants reported higher levels of engagement when training with the SAR. Given that the architecture has been proven to be successful for a rehabilitation task, other sets of repetitive tasks could also take advantage of the platform, such as clinical tests. A commonly performed clinical trial is the Timed Up and Go (TUG), where the patient has to stand up, walk 3 m to a goal line and back, and sit down. To handle this test, we extended the architecture to evaluate lower limbs’ motions, follow the participants while continuously interacting with them, and verify that the test is completed successfully. We implemented the scenario in Gazebo, by simulating both participants and the interaction with the robot^3^. A full interactive report is created when the test is over, providing the extracted information to the specialist. We validate the architecture in three different experiments, each with 1,000 trials, using the Gazebo simulation. These experiments evaluate the ability of this architecture to analyse the patient, verify if they are able to complete the TUG test, and the accuracy of the measurements obtained during the test. This work provides the foundations towards more thorough clinical experiments with a large number of participants with a physical platform in the future. The software is publicly available in the assistive-rehab repository^4^ and fully documented.

## 1 Introduction

According to the World Health Organization (WHO)^5^,^6^ (WHO, 2015), the percentage of healthcare dependent population, such as elderly and people with disabilities, among others, will increase over the next years. This trend will overwhelm the health and social systems of most countries. The adoption of robots could assist these health systems in responding to this increased demand, particularly in high intensity and repetitive tasks.

Most countries, particularly first world countries, are faced with an increasingly higher percentage of people in need of healthcare assistance, either due to old age or some disabilities or impairments, requiring prolonged care, constant examination, and following up the patients very closely. This pressure, both in number of and time dedicated to the patients, will put a strain on the current healthcare and social systems all over the world. Solving this problem, however, is not a simple matter, not just due to the lack of professionals but also due to the increasing costs of maintaining these services. In this paper we propose a solution by using robots to assist medical professionals when realizing repetitive and monotonous exams, in particular for the Timed Up and Go (TUG) test. The implementation of such a system would not only remove some of the workload from the medical professional, but also be more reliable in the long run, not suffering from attention, exhaustion or other issues that often affect us. With this system we intend to show the possibility of using robot platforms in this context, paving the way for further development and implementation of such platforms in hospitals and other healthcare facilities.

The task described in this paper, TUG, is a common screening test performed on patients recovering or suffering from impaired locomotion (Podsiadlo and Richardson, 1991). In this test the patient starts from a sitting position and is asked to stand up, walk to a marker and back, and sit down again. The time the patient takes to complete the tests, along with other metrics like cadence, are used as indicators to evaluate the patient’s static and dynamic balance as well as the mobility. The goal of the robot platform is, in this case, to guide and monitor the patient in the execution of the task, meanwhile retrieving and recording these metrics. The data can then be checked by the medical professional for evaluation, and a more accurate log can be kept to evaluate the recovery of the patient over time. The long-term vision is the development of a robotic solution that takes care autonomously of all the activities involved in the administration of the TUG test in an hospital setting: engaging the patients in their rooms, leading them to the testing room, explaining the test and interacting with the patient to answer questions, monitoring the execution of the test and correcting the patient if needed, and finally sending the results of the test to clinicians.

The platform used in the experiments presented in this paper is a R1 humanoid robot (Parmiggiani et al., 2017), developed by Istituto Italiano di Tecnologia (IIT). This platform, which we will call Socially Assistive Robot (SAR), is equipped with both standard color cameras and with depth cameras to better track the patient. It relies on wheels for navigating in the rooms, using a infrared laser (LIDAR) for obstacle avoidance. Finally, it is equipped with face expressions and speakers to afford interaction with the patients.

The SAR was previously compared to a Virtual Agent (VA) during rehabilitation tasks, with the SAR reporting a higher level of engagement from the patients (Vasco et al., 2019). To develop the automated version of the TUG with the SAR, we extended the architecture to evaluate lower limbs’ motions, follow the participants while continuously interacting with them, and verify that the test is completed successfully. In order to validate the framework and assess its robustness, we implemented the scenario in the simulation environment Gazebo, by simulating both participants and the interaction with the robot.

With this paper we aim to introduce a robotic solution for the increasing strain on healthcare systems, allowing repetitive tests and exams to be handled by a robot platform, removing some of the pressure from the medical professionals, preventing errors due to exhaustion and stress, and maintaining consistency over time and across patients. The aim is to prove the ability of the architecture to assist a therapist in evaluating patients, extracting consistent and reliable data in terms of time, number of steps and the success or failure of the test by the patient. This work is the first step towards more thorough clinical experiments with a large number of subjects with the physical platform.

This paper is structured as follows: in Section 2 we present some previous works on the use of robotics and other devices in healthcare and rehabilitation robotics. In Section 3 we describe in detail the TUG test, and the SAR and the software architecture responsible for the test, along with the description of the test and the data collected. In Section 4, we describe the design of the experiments and the extraction of the ground truth. In Section 5 we present the results of the quantitative analysis of the architecture in simulation. Section 6 discusses the quantitative results, proposing possible improvements to architecture. We conclude the paper with Section 7 where we present our conclusions and discuss further improvements and other tests that could be handled by such a SAR.

## 2 Related Work

The field of robotics in healthcare has been rapidly evolving in the last years, leading to a substantial paradigm shift. An example of this is the Da Vinci system, a surgical robot tele-operated remotely, acting as guidance tool to simultaneously provide information and keep the surgeon on the target (Moran, 2006). This type of robots has been installed and used worldwide. The use of robots for surgery has given rise to a large number of applications for use in the medical domain.

In the particular sub-field of Assistive Robotics, robots are endowed with the human capabilities to aid patients and caregivers. The robot RIBA (Robot for Interactive Body Assistance) is designed with the appearance of a giant teddy bear to lift and transfer patients from a bed to a wheelchair and back (Mukai et al., 2011). The caregiver can instruct the robot through vocal commands and tactile guidance: the desired motion is set by directly touching the robot on the part related to the motion. A similar system, RoNA (Robotic Nursing Assistant), executes the same task, but is instead controlled by the operator through an external GUI (Ding et al., 2014). Chaudhary et al. (2021) plan to design a new medical robot, called BAYMAX, which will serve also as personal companion for general healthcare. The robot will be equipped with a head, comprising a camera, microphone and speakers. It will also contain a series of sensors to detect the temperature, the heartbeat and the oxygen level, and will be capable of performing regular basic check-ups, such as temperature, oxygen level check, mask verification, external injuries etc.

Assistive robots can also aid patients through social interaction, rather than offering physical support (Feil-Seifer and Mataric, 2005): these are known as Socially Assistive Robots (SAR). The robot’s embodiment positively affects the users’ motivation and performance, through non-contact feedback, encouragement and constant monitoring (Brooks et al., 2012; Li, 2015; Vasco et al., 2019). The Kaspar robot is a child-sized humanoid designed to assist autistic children in learning new social communication skills, while improving their engagement abilities and attention (Wood et al., 2021). The Bandit robot consists of a humanoid torso, developed by BlueSky Robotics, mounted on a Pioneer 2DX mobile base. It has been used for engaging elderly patients in physical exercises (Fasola and Matarić, 2013) and providing therapies to stroke patients (Wade et al., 2011). Szücs et al. (2019) propose a framework which allows the therapist to define a personalized training program for each patient, choosing from a set of pre-defined movements. The software has been developed on the humanoid NAO robot controlled through vocal commands via Android smartphone interface. The same robot has been used in combination with a virtual environment for a rehabilitation task: the patient replicates the movements shown by the robot while visualizing himself inside a gamified virtual world (Ibarra Zannatha et al., 2013). The robot coaches the rehabilitation exercising, while encouraging or correcting the patient verbally. Lunardini et al. (2019), as part of the MoveCare European project, also propose to combine a robot and virtual games, with the aid of smart devices (e.g. smart ball, balance board, insoles) to monitor and assist elderly people at home. A similar combination can be seen in the work of Pham et al. (2020) within the CARESSES project, where the authors integrate a Pepper robot with a smart home environment in order to support elderly people.

Another example of coaching is the work by Céspedes Gómez et al. (2021) and Irfan et al. (2020), where a study lasting 2 years and 6 months using a NAO robot to coach patients in cardiac rehabilitation proved that patients were more engaged, and generally finished the program earlier, than the ones not followed by a robot. In this work the robot was the means of interaction, while data was collected through multiple sensors, both wearable and external. In particular, in the case study presented in Irfan et al. (2020), the system was instrumental in detecting a critical situation where a patient was not feeling well, alerting the therapists and leading to medical intervention.

Previous research has focused on automating the Timed Up and Go using sensors of various modalities or motion tracking systems. Three-dimensional motion capture systems have been used to measure the walking parameters with high reliability (Beerse et al., 2019). They currently represent the gold standard (Kleiner et al., 2018), but because of their cost, scale and lack of convenience, it is difficult to install these devices in community health centers. Wearable sensors based on Inertial Measurement Units (IMUs) have been extensively used in instrumenting the TUG test for their low cost and fast assessment. They have proved to be reliable and accurate in measuring the completion times (Kleiner et al., 2018). However, they require time-consuming wearing and calibration procedures that cannot usually be performed by the patients themselves, especially by those with motor limitations. Moreover, the possibility to accurately evaluate the movement kinematics, in terms of articular joints angles, through the data extracted from IMU is still under debate (Poitras et al., 2019). Ambient sensors, including temperature, infrared motion, light, door, object, and pressure sensors, have also shown promise as they remove the need to instrument the patient. Frenken et al. (2011) equipped a chair with several force sensors to monitor weight distribution and a laser range scanner to estimate the distance the subject covers. However, this system is relatively expensive, requires specialized installation and has limited range of use. Video data have also been heavily explored, as they are minimally invasive, require little setup and no direct contact with the patient. Several works have adopted Kinect sensors and their skeleton tracking modes (Lohmann et al., 2012; Kitsunezaki et al., 2013) and webcams (Berrada et al., 2007). A more thorough analysis of the application of these technologies to the TUG test can be seen on the review by Sprint et al. (2015).

## 3 The Framework

To develop the proposed framework, we used the humanoid robot R1 (Parmiggiani et al., 2017) and devised a set of modules interconnected on a YARP network (Metta et al., 2006), as shown in Figure 14.

### 3.1 The Timed up and Go

**FIGURE 1:**
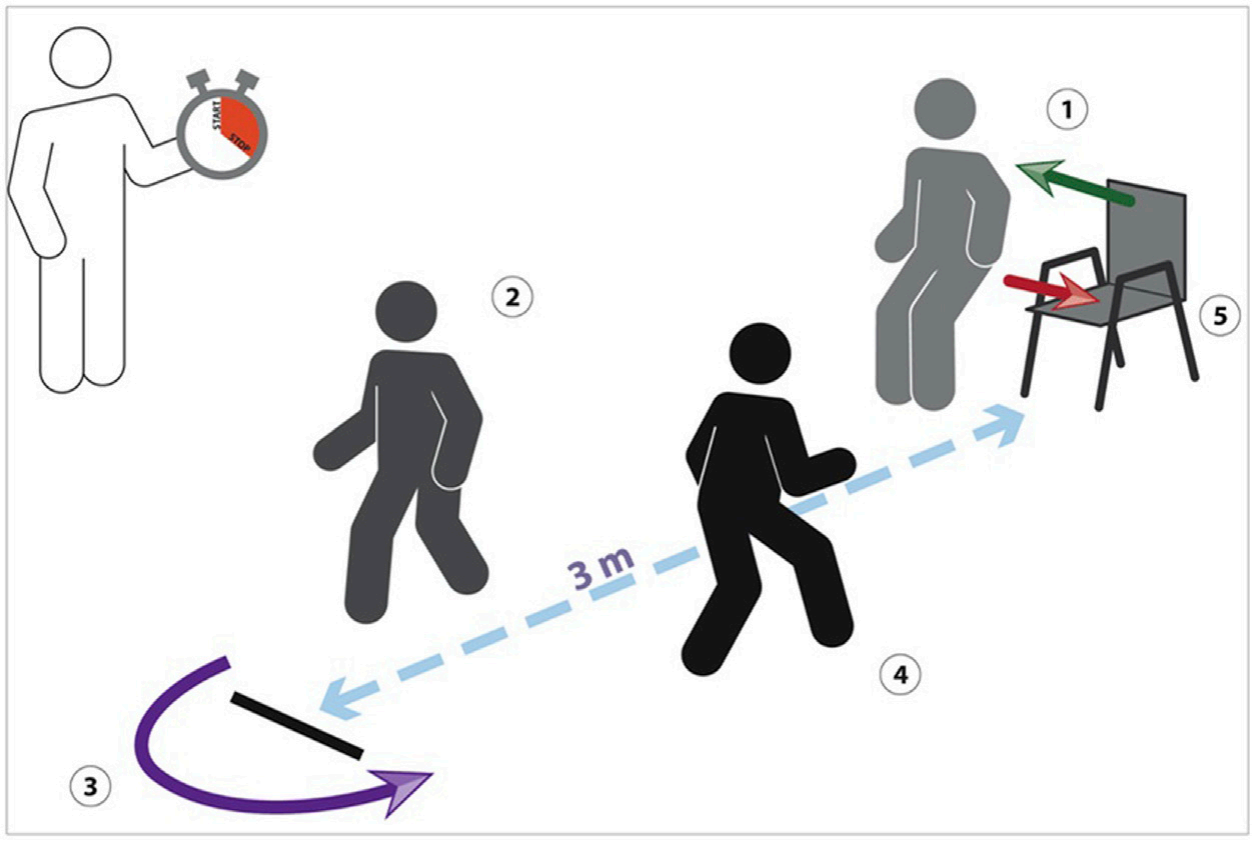
The classical TUG, where the therapist measures the time taken by the patient to perform the task.

The Timed Up and Go is a well-known clinical test widely adopted by clinicians to identify mobility and balance impairments in older adults (Podsiadlo and Richardson, 1991). The test requires the subject to 1) stand up from a chair, 2) walk for 3 m at a comfortable pace, 3) cross a line placed on the floor, 4) go back to the chair and 5) finally sit down, as shown in Figure 1. In typical scenarios, the therapist measures, by means of a stopwatch, the time taken by the patient to perform the task and assess the progress achieved throughout the rehabilitation. Gait parameters such as step length and walking speed are subjectively observed, but not quantitatively measured. Moreover, evaluating if the line was crossed is not always straightforward, especially when the patient approaches it and then turns back.

We extend the architecture previously presented for a rehabilitation task (Vasco et al., 2019) in order to also cover clinical tests and identified the TUG as use case. The automated version of the TUG foresees the execution of the test, while the robot engages the patient in a verbal interaction to illustrate the instructions and answer potential questions, evaluates the movement and verifies if the test is performed correctly.

### 3.2 The Humanoid R1

R1 is a 1.2 m humanoid robot designed with the sensory and actuation capabilities to interact with a dynamic environment. The robot is equipped with two 8DOF arms, capable of elongating when needed, and two 4DOF hands. The torso includes a mechanism that allows it to vary its height from a minimum of 1.15 m to a maximum of 1.35 m. The robot navigates the environment by means of two driving wheels and is equipped with two front and rear LIDAR laser sensors integrated into its base. A curved RGB LED display is mounted into the head, allowing facial expressions. It is equipped with an Intel RealSense depth camera, which provides RGB images along with depth data. Finally, the robot has a speaker and a microphone to acquire sound signals.

### 3.3 Line Detection

The finish line used in the TUG to indicate the end of the path is identified by means of an ArUco marker board. We additionally introduced an ArUco start line, to provide a robust *static* reference frame for the test session. We use the start line pose as reference frame to express the skeletons, the robot and the finish line in such reference frame. In the simulated world, the line positions are pre-determined in the Gazebo scenario, maintaining the same conditions as the real world. The line reference frame is defined with *x* along the line length, *y* pointing backward and *z* pointing upwards.

### 3.4 3D Skeleton Acquisition

The skeleton acquisition is performed by combining the detection of 2D key-points with the related depth. More specifically, yarpOpenPose is responsible for estimating human poses based on OpenPose(Cao et al., 2019), an open-source library for real-time multi-person 2D pose estimation: the module processes an RGB image and outputs a list of 2D key-points for each person found in the image, achieving high accuracy and speed regardless of the number of people inside the scene. skeletonRetriever combines the 2D locations with the depth provided by the camera sensor, to reconstruct key-points in the 3D world, adopting the classical pinhole camera model. The 3D reconstruction of the skeleton is guaranteed to be robust against keypoints self-occlusions and mismatches occurring between detected keypoints and noisy depth contours (Gago et al., 2019).

### 3.5 Motion Analysis

The motion analysis component extracts in real-time metrics relevant for the TUG. More specifically, motionAnalyzer is responsible for estimating the Range of Motion (RoM) of the articular angles and the gait parameters, i.e. number of steps, step length and width and walking speed. More in detail, we compute the distance between left and right foot and detect a step each time such distance reaches the maximum value. The step length and width are then measured by projecting the maximum feet distance along the skeleton sagittal and coronal planes respectively. Finally we estimate the walking speed as the product of the step length and the number of steps over the time taken to complete the test.

The module is also in charge of computing measurements relevant to establish if the patient has passed the test:

• the distance between the skeleton hip center and the finish line along the y coordinate, in order to identify if the line was crossed. A “crossed line” event occurs if a change of sign is detected in the estimated distance;
• the neck speed along the z coordinate to identify if the patient has stood up (and thus started the test) and sat down (and thus completed the test). A “standing/sitting” event occurs if the neck speed is higher/lower than zero (i.e. the neck height increases/decreases).

The module additionally exports relevant data for enabling offline reporting of the experiments at the end of the session. The aim is to provide the clinician with a tool for evaluating offline the quality of the movement and use it as documentation that can be added to the patient clinical report.

### 3.6 Speech Interaction

The robot explains the task to the patient, providing pre-defined verbal instructions through its speaker. The platform is also able to reply to a set of potential questions, using two interconnected layers relying on the Google services API, which have proved to be very robust especially with the Italian language (Calefato et al., 2014): googleSpeech, in charge of converting the sound provided by the microphone into transcript, and googleSpeechProcess, in charge of analyzing the speech transcripts in order to retrieve the sentence structure and meaning. Such system is constrained to a set of pre-defined topics related to the TUG, each associated to a pre-defined answer provided by the robot. Two are the potential sources of error: 1) the sound is not understood (at googleSpeech level) and 2) the question does not belong to any of the handled topics (at googleSpeechProcess level). In the first case, the robot asks the patient to repeat the question, whereas in the second case, it informs the patient that the question does not belong to its known repertoire and cannot reply to it. The association between topic and related answer is listed in Table 1. The system is capable of interpreting the question, rather than recognizing it, providing a flexible and natural interaction. Therefore, a question related to the same topic can be posed in several different ways, in a natural language, rather than using vocal commands.

**TABLE 1:** The list of topics and related answer provided through verbal interaction.

Topic	Answer
Speed	“You can move at your normal speed.”
Feedback	“You are moving very well/well/not very well.”
Aid	“You can use the walking aid that you need.”
Repetitions	“You have to repeat the test once.”
Unclear	“I didn’t understand the question. Can you please repeat it?”
Unknown	“I’m sorry. I don’t know how to answer to this question.”

A WiFi button has been integrated in the robot infrastructure: when pressed, it triggers the speech pipeline. Such trigger is useful to avoid the system to be always responsive, and thus sensitive to background noise.

### 3.7 Reactive Navigation

The reactive navigation system allows the robot to navigate the environment based on the received perceptual stimuli. More specifically, navController commands the robot wheels to navigate along an imaginary straight path while keeping a fixed distance from a specified skeleton. Such distance is designed in order to maximize the observability of the body key-points and guarantee the whole skeleton to be within the cameras field of view. Given the desired distance from the target skeleton, we designed a simple bang-bang controller which commands the robot speed using as feedback the distance between the robot location provided by the odometry and the skeleton location provided by the vision pipeline. A further command modality is available to allow the robot to reach fixed points in the environment (e.g. start and finish lines adopted in the TUG).

Obstacle avoidance was additionally implemented, which clusters data provided by the lasers and stops the navigation if the robot reaches the closest obstacle. In such case, the robot asks for removing the obstacle and the interaction is suspended until the obstacle is removed.

### 3.8 The Gazebo Actor

We simulated the patient executing the TUG resorting to the Gazebo animated model, called Actor. Actors extend common models with animation capabilities, by combining the relative motion between links and the motion of all the links as a single group along a trajectory. For developing the virtual TUG, we relied on the animations provided by Gazebo to stand up, sit down and walk defined within COLLADA files. We customized the walking trajectory by specifying the poses to be reached at specific times, in order to allow the Actor to walk 3 m and then go back. We also developed a Gazebo plugin to play/stop the animation, update the walking speed and reach specific targets in the environment.

The software is publicly available in the assistive-rehab repository^7^ and fully documented. The dedicated website^8^ also provides an overview of the work, including tutorials for running the software.

## 4 Experimental Design

**FIGURE 2:**
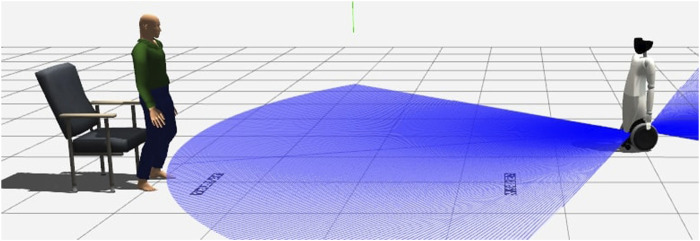
The TUG developed in Gazebo.

In order to test the architecture, we developed a simulation environment in Gazebo containing an Actor representing the human patient, the robot platform, the two ArUco markers and a chair. The scenario can be visualized in Figure 2. This environment allows the intensive testing required to perform a first quantitative validation of the software. The main objective of these experiments is to verify the accuracy of the robot when compared to a ground truth that we obtain from the environment itself. In this section we will discuss how to obtain the ground truth from the environment, along with the three experiments we perform in these conditions.

For each trial, we first set the conditions of the trial (e.g.: speed, target distance) for the Actor. We then compute the number of steps the Actor should take to complete the trial, while updating the odometry of the SAR platform to ensure accurate repetition of the experiment. We then start the trial, measuring the true time the Actor takes to complete the trial. Finally, we store all the data for this trial. We performed 1,000 trials for each of the experiments, in order to provide enough data for a significant quantitative validation.

### 4.1 Ground Truth

To verify the system, we need to compare its measurements with the actual situation in the scenario. The data acquired by the robot consists of the time to complete the test, the number of steps the patient takes, and whether they passed the test or not. Retrieving this information from Gazebo simulation is not trivial, since there is no particular structure we can collect this data from. We must, therefore, compute these values for each trial.

The time to complete the experiment consists of the time between the user first standing up and then finally sitting down. The Gazebo Actor performs actions based on a set of animations, and it is possible to query the current animation being played. The time is calculated between the state transitions “sitting - stand up” and “standing - sit down”. Unfortunately, the time of the standing up and sitting down animations is not constant between trials, so we had to measure the time for each trial individually, even when the conditions were otherwise unchanged.

To obtain the true number of steps we computed how many steps the Actor would have to take to reach the desired target and return, adding one extra step for the turn. This is possible since the step length of the Actor is fixed.

**FIGURE 3:**
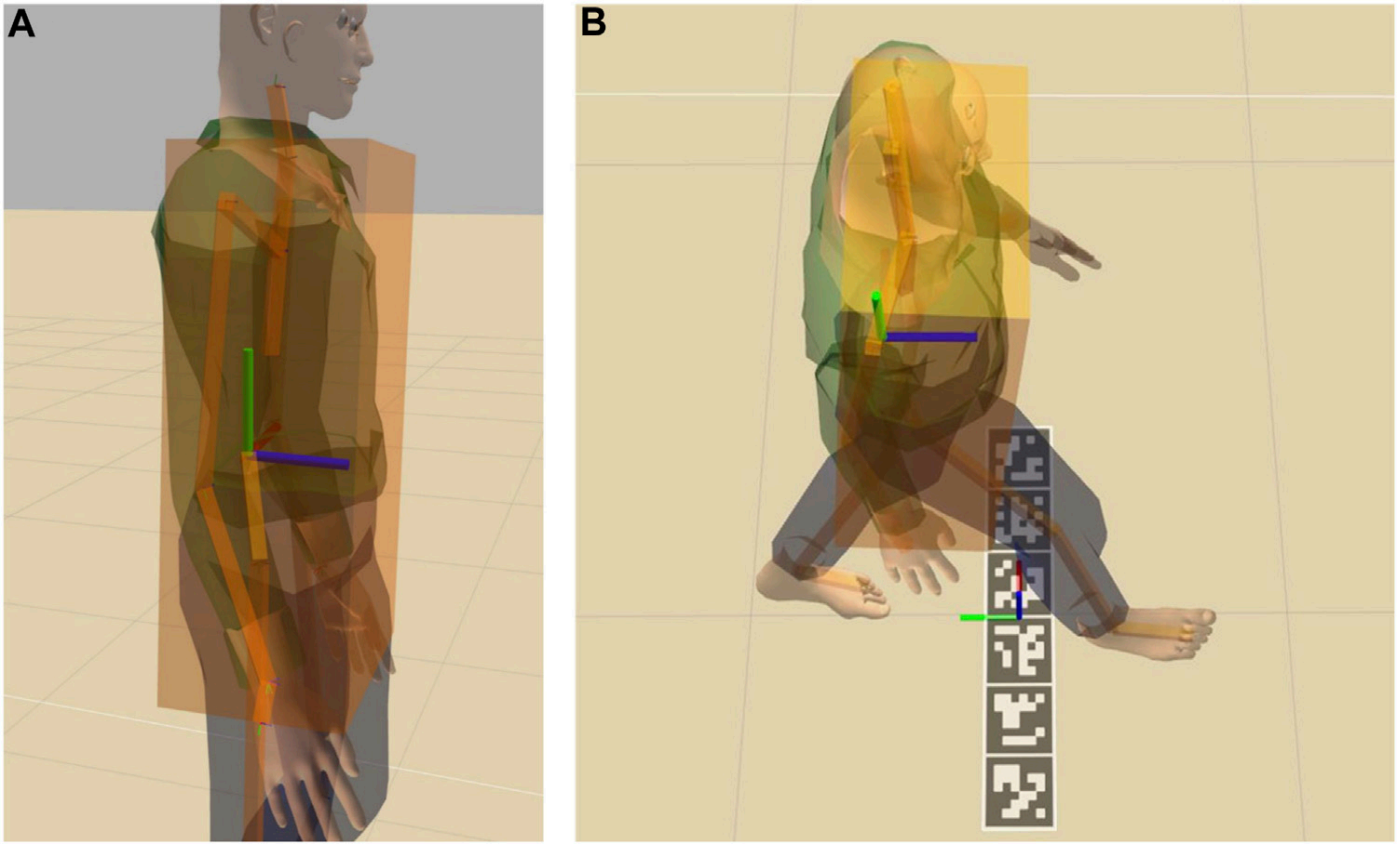
The Actor with the bounding box around the torso and the lower spine keypoint **(A)** and the Actor walking compared to the finish line frame **(B)**. Red green and blue axes represent x, y and z respectively.

Finally, to verify if the Actor passed the test, we had to verify when it crossed the target line. The Actor’s animated motion is defined with respect to a keypoint placed in the lower spine. Since the tracking is performed at the waist level instead, and the position is obtained by measuring the position with depth cameras, we had to verify when the front of the waist crossed the line instead of relying on the center of the Actor. To compute this, we fitted a bounding box around the Actor’s torso and calculated the offset of the front surface of the waist of the Actor in relation to its center, as shown in Figure 3. We could then calculate if the Actor passed or not a test simply by checking *actorTarget* + *offset* > = *testTarget*. For example, Figure 3B compares the use of the Actor lower spine with the bounding box when the Actor crosses the finish line: if the front surface of the waist is not considered, the ground truth for this trial would erroneously be classified as not passed.

### 4.2 Experiment Conditions

Each experiment tested a different aspect of the system, in order to validate its accuracy under different conditions.

In experiment 1 we changed the speed of the Actor in order to test how good the tracking of the system performed under different speeds. Each trial we sampled the speed from a uniform distribution between 0.5 and 1.3, corresponding to roughly the same value in meters per second. We chose these values considering the average walking speed of the elderly patients, which is typically around 0.8–0.9 m/s (Busch et al., 2015). No other conditions were changed in the trial, with the Actor having to move 4 m forward (4.5 m from origin of the simulation), turn around, and return to the original position. The finish line is set at 3 m from the start (3.5 m from origin of the simulation) as defined in the TUG test, and we set the target distance to 4 m in order to guarantee that the Actor crosses the line. The number of steps of the Actor were thus unchanged for every trial, while the time changed based on the speed.

In experiment 2 we changed instead the target distance for the Actor. With this experiment we studied how the system reacted to a different number of steps. For each trial the target position for the Actor was sampled between 2.0 and 5.0 m (2.5–5.5 m from the origin of the simulation). The speed was set constant at 0.9. In this trial both the time and number of steps varied.

Finally, in experiment 3 we studied the accuracy of the system when detecting if the patient passed the test or not. To verify this, we again sampled the target distance of the Actor but around a much narrower window, between 3.0 and 4.0 (3.5 and 4.5 m from origin of the simulation), in order to test the limit condition. As in experiment 2, the speed here is set constant at 0.9 for all the trials. The main target of this experiment is to verify the pass/not-pass result.

## 5 Results

### 5.1 Experiment 1: Changing the Actor’s Speed

**FIGURE 4:**
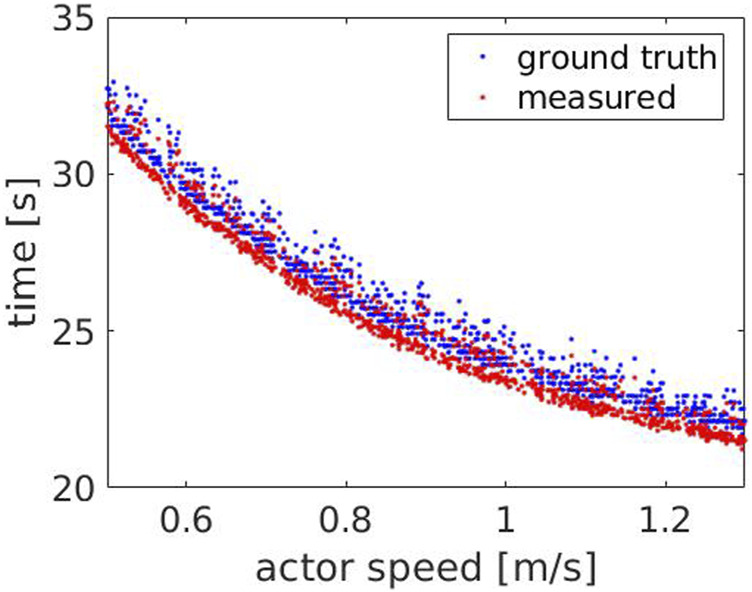
Experiment 1: the measured (red) and the ground truth time (blue) with respect to the Actor’s speed.

Figure 4 shows the time measured by the robot compared to the ground truth time, with respect to Actor’s speed. The result indicates that the measured time consistently follows the ground truth, decreasing for increasing speeds, with an average error of $\left[0.47\pm 0.06\right]s$ . Such error occurs due to an inherent limitation in the definition of the ground truth time: we start measuring this time when the stand up animation starts, which includes also an initial part where the Actor sits. Similarly, we stop measuring the ground truth time when the sit down animation stops, which includes a final part where the Actor sits. The robot instead *detects* if the Actor is about to stand or sit based on the neck position. Therefore, it does not take into account the additional contribution provided by the ground truth, resulting in lower values.

**FIGURE 5:**
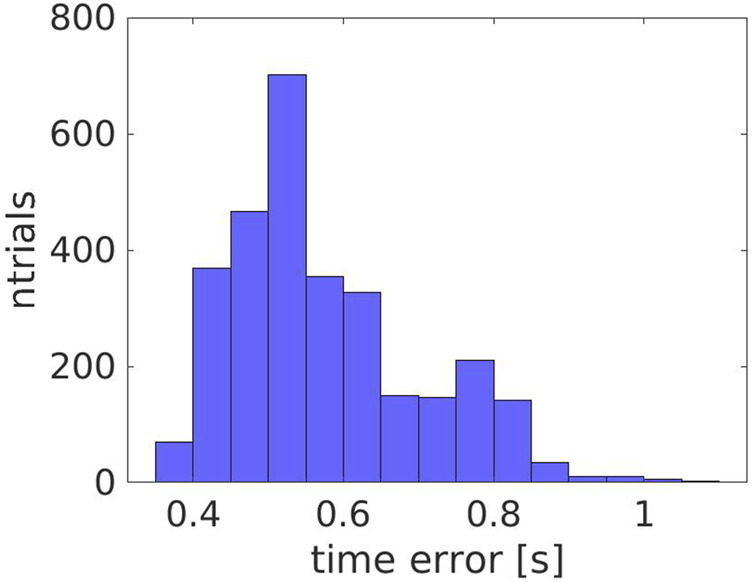
The error in time for all the experiments (1, 2 and 3).

In order to evaluate the consistency of the error for different scenarios, we estimated it over all the experiments, 1, 2 and 3. As shown in Figure 5, the error is coherent with an average value of $\left[0.77\pm 0.86\right]s$ .

**FIGURE 6:**
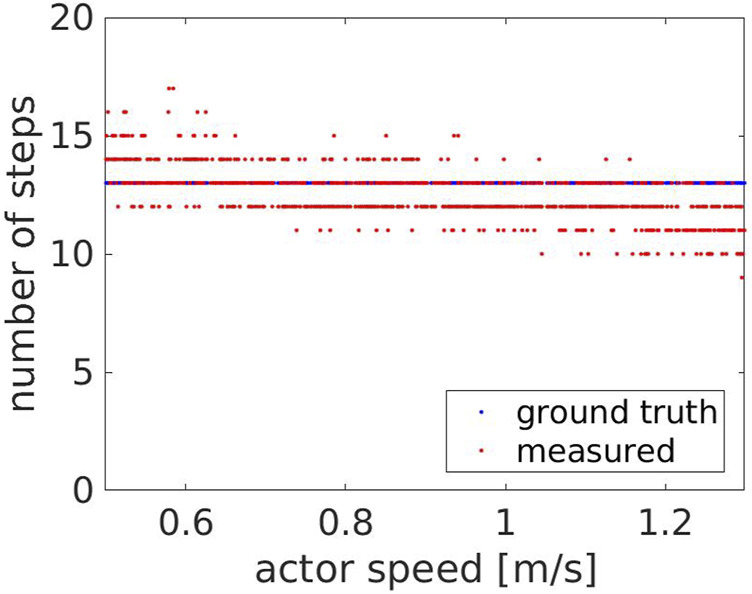
Experiment 1: the measured (red) and the ground truth number of steps (blue) with respect to the Actor’s speed.

**FIGURE 7:**
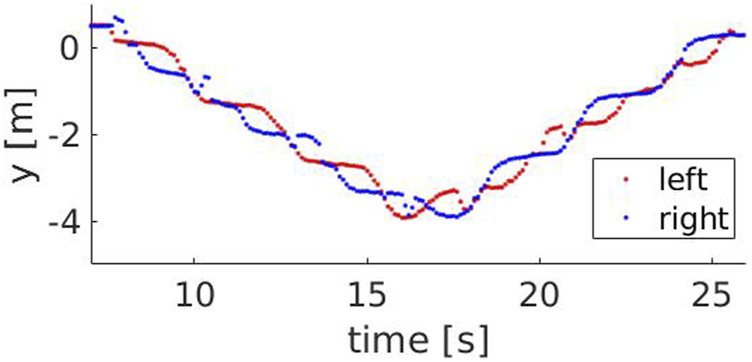
The y component of the left (red) and right (blue) ankle for a speed of 0.9 m/*s*.

**FIGURE 8:**
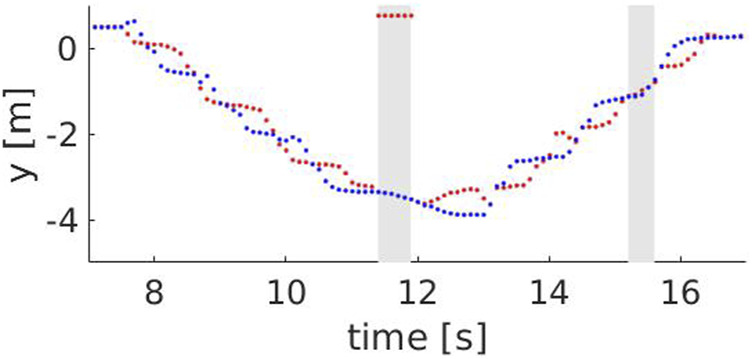
The y component of the left (red) and right (blue) ankle for a speed of 1.3 m/*s*. The grey areas show time intervals where steps are missed, i.e between 11.4 and 11.9 s (skeleton falling out of the field of view) and between 15.2 and 15.6 s (skeleton is too far from the robot).

**FIGURE 9:**
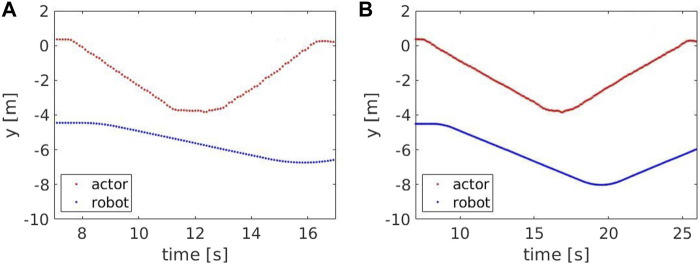
The y component of the Actor’s hip center (red) and robot base (blue) for a speed of 1.3 m/*s* **(A)** and 0.5 m/*s* **(B)**.

For the speeds evaluated in this experiment, we compared the number of steps measured by the robot with the ground truth, which was estimated to be 13 steps. As shown in Figure 6, the measured number of steps (red dots) follows the ground truth (blue line), with an average error of $\left[0.64\pm 0.71\right]$ steps. For higher speeds, the probability of underestimating the number of steps is higher. Figures 7, 8 show the y component of the left and right ankles for two different speeds, 0.9 and 1.3 m/*s* respectively. The walking pattern is clear for both speeds, with the left and right ankles alternating and then switching when the Actor turns back. However, at higher speeds, some steps are missed when the Actor approaches the robot (between 11.4 and 11.9 s) or when he moves away from it (between 15.2 and 15.6 s). This occurs because the robot maintains a fixed distance from the Actor’s hip center, but is unable to keep it when their speeds are considerably different. Figure 9 compares the Actor (red) and the robot locations (blue) when the Actor walks at 0.6 m/*s* (A) and 1.3 m/*s* (B) respectively and the robot moves at 0.4 m/*s*: when the speeds (i.e. the slopes) are comparable, the distance between the curves is fixed, indicating that the robot can properly follow the Actor. However, when the speeds start deviating significantly, the distance between the curves varies with two potential detrimental effects: 1) the skeleton partially falls out of the field of view and thus some steps are missed and 2) when the Actor turns back, the robot moves away from it to reach the pre-defined distance. This can lead to the mis-detection of steps in the 2D image.

### 5.2 Experiment 2: Changing the Target to Reach

**FIGURE 10:**
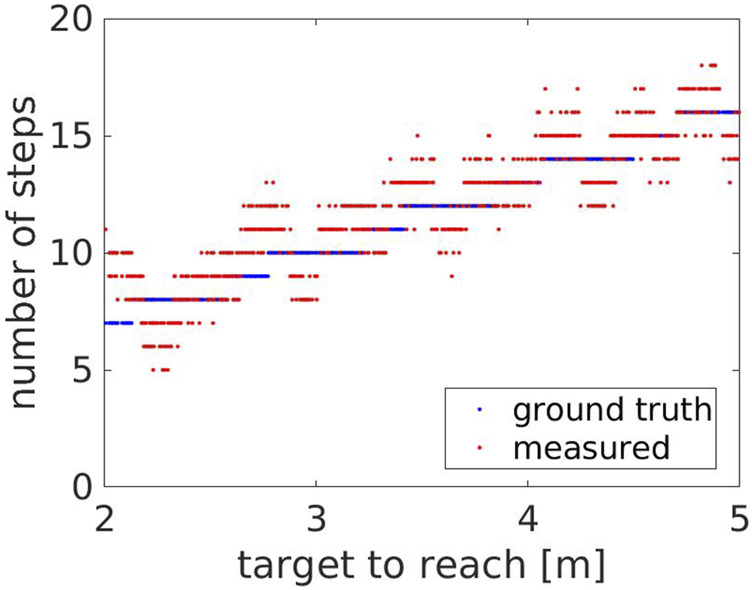
Experiment 2: the measured (red) and the ground truth number of steps (blue) with respect to the target to reach.

**FIGURE 11:**
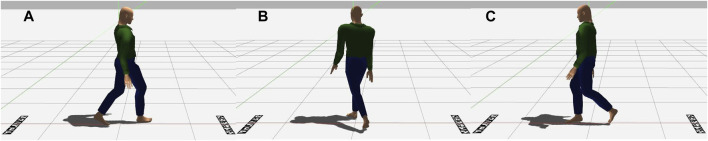
The Actor turning back when reaching a target distance at 2.0 m.

**FIGURE 12:**
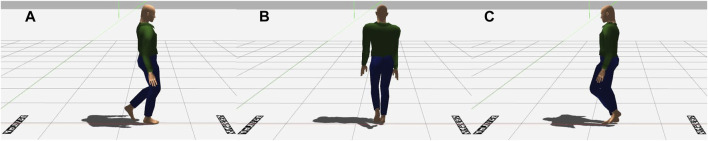
The Actor turning back when reaching a target distance at 2.3 m.

Figure 10 shows the number of steps measured by the robot compared to the ground truth steps, with respect to the target to reach. The measured number of steps consistently follows the ground truth, increasing for increasing distances, with an average error of 1.11 ± 0.80 steps. However, around the transition areas corresponding to steep changes in the ground truth steps, the measured number of steps is over- and under-estimated respectively before and after the change. This occurs as the step’s length the Actor does when turning back changes with the target distance. Figures 11, 12 show the Actor turning back when reaching a target distance of 2.0 and 2.3 m respectively. In the first case, the step is wide and measured twice, both when the Actor moves forward and then back. In the second case, the step instead is narrower and not detected by the robot. The same effect applies when the Actor reaches the chair and turns around to sit down. However, this is an artifact of the Actor animation, as it rigidly turns around its center of mass.

### 5.3 Experiment 3: Evaluating the Accuracy Around the Finish Line

We computed the percentage of true positives/negatives, false positives/negatives and evaluated accuracy, precision and recall. We achieved an accuracy of 92.30*%*, with a precision of 90.09*%* and a recall of 100.0*%*. These results were obtained very close to the finish line, where false positives/negatives are more likely to happen, a worst case scenario for this type of measurement; on regular tests, this type of error is even less likely to happen.

**FIGURE 13:**
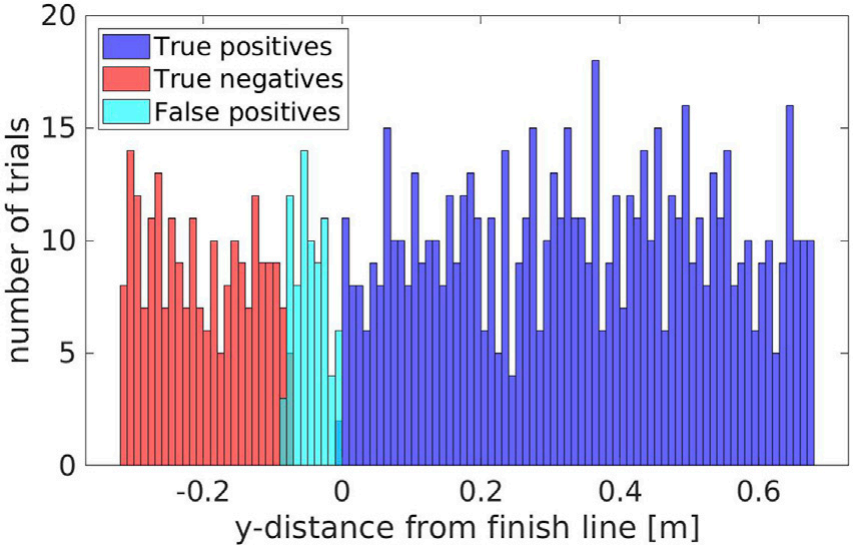
The amount of true positives/negatives and false positives/negatives grouped with respect to the y-distance from the finish line.

We can visualize this effect on Figure 13, where the false positives are all concentrated just before the finish line, and in very low number. We can also see that the area where false positives are detected, within 0.08 m from the finish line, we have only false positives. The absence of true negatives in the same area suggests that the false positives are not due to noise in the measurement of the skeleton, but rather a minor offset between the skeleton detected by the robot and the ground truth estimated for the Actor waist. In any case, this offset is in the order of centimeters, and should not affect the overall trial. No false positives nor false negatives were detected beyond the range shown in Figure 13.

## 6 Discussion

With the results of the experiments discussed in section 5 we tested the effectiveness of a robot architecture to evaluate a TUG test with different patient speeds and distances. The system performed well, keeping track of the patient and accurately measuring the relevant data, namely time and number of steps, with which we can estimate cadence, step length, speed.

The measurement of the time of the trial is accurate in all the range of Actor speed, keeping a consistent error throughout the trials which, as explained in Section 5.1, is due to a discrepancy between the Actor state of sit/standing and the measurement of the robot, which relies on a particular height threshold. Future improvements could fine-tune the system to account for this error by calibrating the threshold based on the height of the patient.

The number of steps measured is also quite accurate, particularly considering the speed range of the target patients, consisting of elderly and motion-impaired patients. The average error of 1.1 ± 0.80 steps is not negligible compared to a standard TUG execution. However, this is widely due to artifacts of the Actor’s animated motion when turning back, as described in Section 5.2, and will potentially improve when testing the architecture on the real platform. The accuracy is also reduced for patients that perform the test with higher speeds, in a big part due to the inability of the robot to follow the patients through the test. Some possible improvements to obtain better results and mitigate the misdetections could be to improve the navigation, by adapting the distance between robot and patient to the measured patient’s walking speed. Finally, improvements in skeleton detection models, particularly when integrating prior information of the human kinematic structure, could also lead to more accurate measured keypoints, which in turn would lead to better estimation of the steps. Also, different skeleton detection methods could be explored (Lugaresi et al., 2019), which directly include depth perception in the skeleton model (Wang et al., 2021).

The accuracy of the success/fail condition of the TUG test was also evaluated, particularly around the finish line, in order to evaluate the ability of the SAR to successfully measure the result of the test. The accuracy of this measurement was quite high and consistent across trials, which could prove to be an advantage when compared to evaluation by a physiotherapist, particularly in cases where it is not entirely clear if the patient passed the test or not. The trials also detected only false positives and no false negatives, and the results suggest an offset is present between the measured skeleton and the ground truth. The overlap between false positives and both true negatives and true positives is also quite small, meaning with some calibration of this measurement to eliminate this offset we could minimize the number of false positives obtained during the trials.

With these results we validate, in simulation, the capability of the system to assist physiotherapists in performing the TUG test, collecting valuable data for analysing the improvement of patients. Further validation, to be performed in future work, comprises execution on the physical platform, running trials with naive participants and collecting performance data. This should provide a more accurate picture of the behavior of the system in the real world.

## 7 Conclusion

**FIGURE 14:**
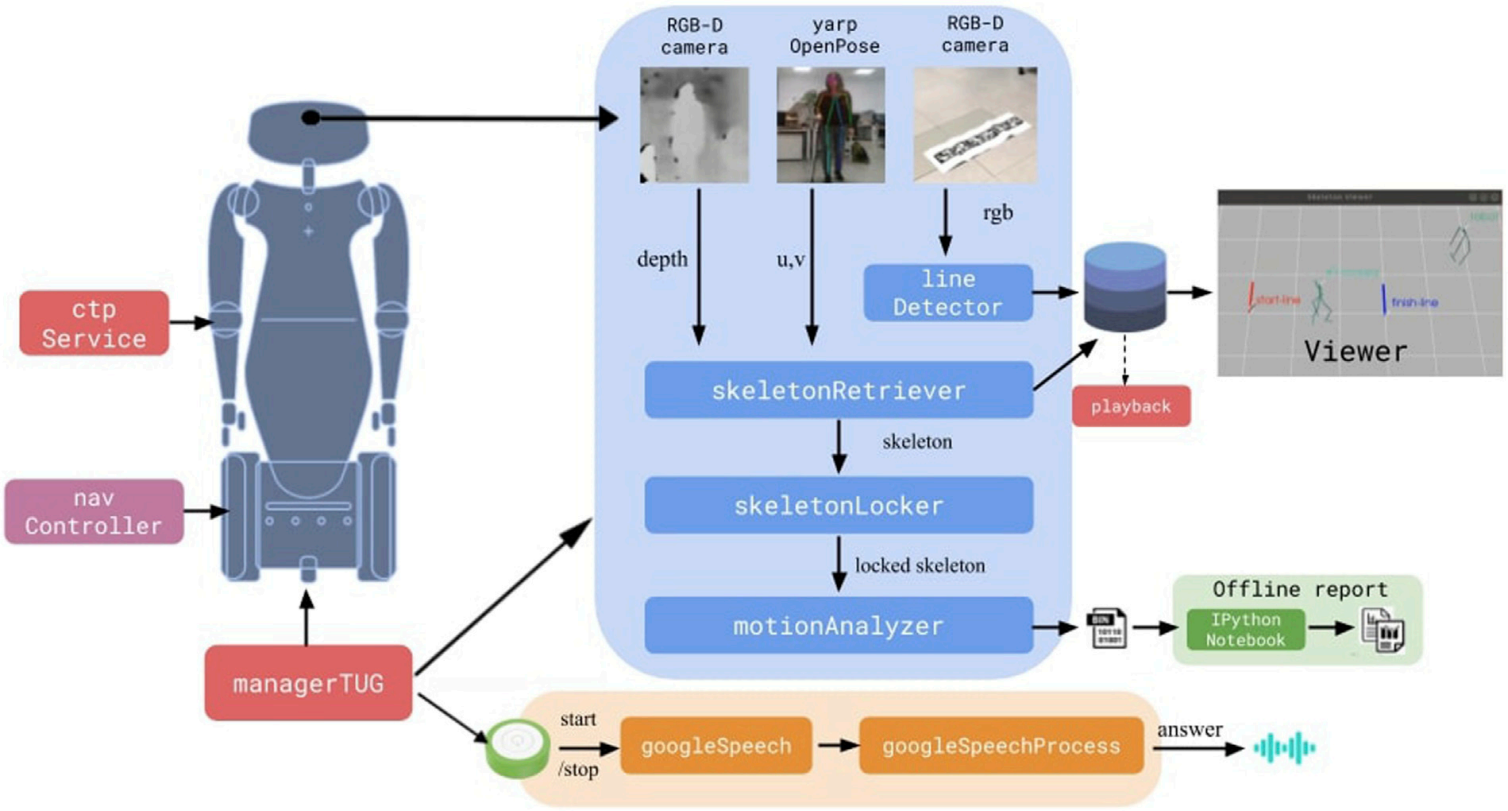
The devised framework.

In this paper we presented a software architecture implemented in an R1 robot that guides and monitors patients while performing a TUG trial. The architecture consists on multiple modules as shown in Figure 14, from navigation and control, to skeleton detection and tracking, to verbal interaction, all integrated by means of YARP. An analysis of its execution was provided using a Gazebo simulation with a simulated Actor. The data extracted during these trials showcase the ability of such an architecture to assist a therapist in evaluating patients, extracting consistent and reliable data in terms of time, number of steps and the success or failure of the test by the patient.

The architecture will be further evaluated and validated on the physical platform, first with non-naive healthy subjects and then with patients in hospital settings. In the long term, we aim at developing a robotic solution that takes care of all the activities involved in the administration of the TUG test in the hospital. Such a system would release the strain on the current healthcare and social systems and yield a positive impact on the cost-effectiveness of rehabilitation pathways.

Possible improvements to the current model were also discussed in Section 6, from improvements in the robot platform to more accurate skeleton depth perception. Additional improvements could be appended to the architecture, in order to make use of the rich amount of data collected during the trials. One such example that could be extremely useful, particularly considering the target patients for this test, who are often motion-impaired and prone to falls, is to detect patterns that lead to falls and raise an alert when such a pattern is detected. This would further help the physiotherapist in preventing serious harm to patients that start displaying falling patterns. Similarly, the system could learn to detect common walking impairments that could go unnoticed without consistent data analysis, thus allowing the physiotherapist to monitor the situation and act accordingly.

Furthermore, the current navigation system is purely reactive, allowing the robot to navigate based on the received perceptual stimuli, coming from front and back LIDARs. Some objects are not detected by these sensors, given their limited field of view and range (e.g., tables). Visual perception could overcome these limitations and complement the use of the LIDARs.

Finally, the speech system used in this architecture is currently quite simple, relying on a simple, if sophisticated, query system responsible for answering patient questions. Further development in this aspect could see the use of a conversational agent, which would enhance the interaction with the patients and leave them more at ease when interacting with the robot and physiotherapist.

## Data Availability

The raw data supporting the conclusions of this article will be made available by the authors, without undue reservation.

## Footnotes

5
https://www.who.int/news-room/fact-sheets/detail/ageing-and-health

7
https://github.com/robotology/assistive-rehab

8
https://robotology.github.io/assistive-rehab/doc/mkdocs/site/
